# A mathematical model of tumor-endothelial interactions in a 3D co-culture

**DOI:** 10.1038/s41598-019-44713-2

**Published:** 2019-06-10

**Authors:** Yamicia Connor, Yonatan Tekleab, Sarah Tekleab, Shyama Nandakumar, Divya Bharat, Shiladitya Sengupta

**Affiliations:** 1 Harvard-MIT Division of Health Sciences and Technology, Cambridge, MA 02139 USA; 2 Massachusetts Institute of Technology, Department of Aeronautics and Astronautics, Cambridge, MA 02139 USA; 3 Brigham and Women’s Hospital, Department of Medicine, Boston, MA 02115 USA; 4 Harvard Medical School, Health Sciences & Technology, Boston, MA 02115 USA; 5 Beth Israel Deaconess Medical Center, Department of Medicine, Boston, MA 02215 USA

**Keywords:** Cytological techniques, Metastasis

## Abstract

Intravasation and extravasation of cancer cells through blood/lymph vessel endothelium are essential steps during metastasis. Successful invasion requires coordinated tumor-endothelial crosstalk, utilizing mechanochemical signaling to direct cytoskeletal rearrangement for transmigration of cancer cells. However, mechanisms underlying physical interactions are difficult to observe due to the lack of experimental models easily combined with theoretical models that *better* elucidate these pathways. We have previously demonstrated that an engineered 3D *in vitro* endothelial-epithelial co-culture system can be used to isolate both molecular and physical tumor-endothelial interactions in a platform that is easily modeled, quantified, and probed for experimental investigation. Using this platform with mathematical modeling, we show that breast metastatic cells display unique behavior with the endothelium, exhibiting a 3.2-fold increase in interaction with the endothelium and a 61-fold increase in elongation compared to normal breast epithelial cells. Our mathematical model suggests energetic favorability for cellular deformation prior to breeching endothelial junctions, expending less energy as compared to undeformed cells, which is consistent with the observed phenotype. Finally, we show experimentally that pharmacological inhibition of the cytoskeleton can disrupt the elongatation and alignment of metastatic cells with endothelial tubes, reverting to a less invasive phenotype.

## Introduction

Mathematical models are useful tools to simplify complex systems in order to better understand physiological dynamics of biological processes. Cancer metastasis is a complex and multifaceted process that involves changes at genetic, mechanochemical, and environmental levels. As a result of this complexity, coupling experimental models with mathematical models presents a robust way to mimic, quantify, and describe tumor behavior. Furthermore, mathematical models can be used to support and validate hypotheses and experimental results. Using mathematical models, we can better understand the factors governing complex processes by stripping them down to the most influential variables. Mathematical and computational models are used widely in cell biology on scales ranging from gene expression to cell population dynamics^1–4^. Several properties intrinsic to the tumor^5,6^, as well as factors governed by reciprocal signaling^7–10^ between the extra cellular matrix and the tumor, have been implicated in increased invasiveness. However, metastasis also involves the interaction between the endothelium and tumor cells. It has been observed that both metastatic cells and the endothelium undergo physical changes that are essential to metastasis. For example, metastatic breast epithelial cells decrease the stiffness of endothelial cells which is believed to enhance epithelial cell transmigration^11^. Similarly, metastatic cells have been found to be 80% more compliant than benign cells, and this decrease in cell stiffness may also enhance the ability of these cells to successfully cross the endothelial cell barrier^12,13^. It is clear that reciprocal interactions between the two cell types is critical for invasion^14^. Successful invasion of metastatic cells into an endothelial vessel requires a coordinated dance of chemical and mechanical signals that control dynamic cellular processes such as cellular membrane extension^15,16^, membrane adhesion, and cellular migration^17^.

Biological processes have been previously described using hyperelastic and viscoelastic models to mathematically characterize the physical behavior of tissue, cells, and biopolymers^18,19^. Characterizing biomaterials as such allows us to create constitutive relationships between physical quantities that can be measured experimentally (e.g. lengths, forces) and quantities we seek to calculate (e.g. elastic moduli, strain energy densities). Numerical simulations have been developed and shown to be consistent with experimental data for these models^20–22^. While the 3D architecture of normal and malignant breast tissues has been well studied in multiple *in vitro* systems^23^, our work aims to extend this research to better understand the physical dynamics between cancer cells and the endothelium by isolating and examining these components in our experimental and mathematical models.

In recognition of the vital cross-talk between tumor cells and the endothelium^24–28^, we engineered a 3D co-culture system combining epithelial and endothelial cells to study endothelial-epithelial cell-cell interactions. In this system, human endothelial cells were allowed to undergo tubulogenesis in a Matrigel® matrix to which normal, non-metastatic, and metastatic breast epithelial cells were added. It is well documented that breast epithelial cells form defined mammospheres when cultured on a 3D matrix^29–32^. These platforms have provided valuable insight into the native cellular morphology and architecture. When breast epithelial cells are co-cultured with preformed endothelial tubes, a unique phenotype develops^28^. Metastatic cells, when in contact with the endothelium, display two unique characteristics: (1) preferential interaction with endothelial vessels and (2) elongation along vessel structures. This is in contrast to normal breast epithelial cells, which have limited interaction with the endothelium and remain in a rounded, non-deformed state. These characteristics can be quantified through two parameters we have developed: (1) epithelial-endothelial association (EEA), which we also refer to as simply *association* and (2) *elongation*. These parameters were developed to quantify the behavior between epithelial cells and the endothelium in our 3D co-culture. For our mathematical analyses, we model the endothelial cell as a neo-Hookean hyperelastic material. This gives us a constitutive relationship to capture the nonlinear cell deformation, allowing for the calculation of tumor cell energy expenditure as it traverses the endothelial tube wall. We demonstrate that highly metastatic epithelial cells have a greater propensity to undergo large deformations because it is a more energetically favorable mechanism of metastasis. The physical characteristics of the epithelial and endothelial cells are obtained from measurements of images of our 3D co-culture. Combining our mathematical and experimental models, we are able to determine that the system expends less energy overall during an endothelium breech when the epithelial cells are able to elongate and align with the endothelium, as is observed more frequently with the phenotypically metastatic cell lines.

## Results

### 3D epithelial-endothelial co-culture captures unique interactions between metastatic cells and the endothelium

**Figure 1:**
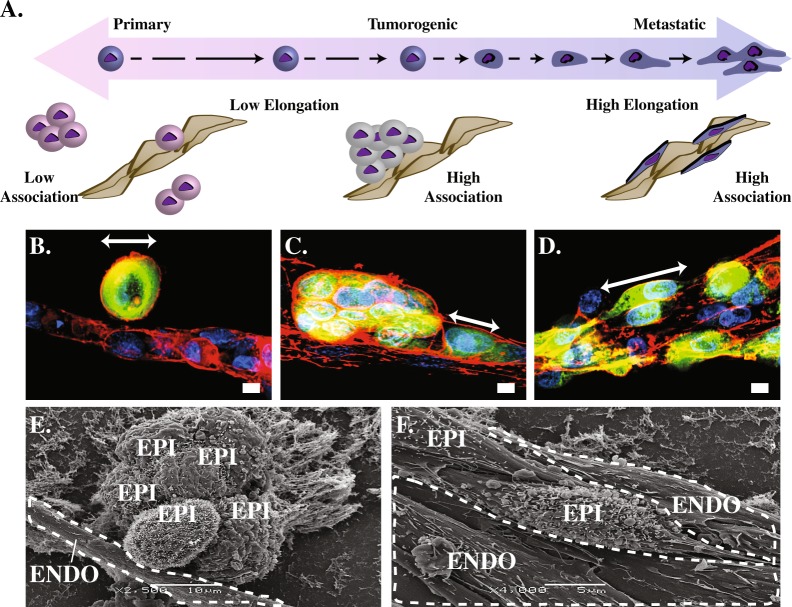
(**A**) Illustration of the range of invasive potential. Non-tumorigenic epithelial cells on the left side; tumorigenic epithelial cells in the center maintaining their rounded phenotype and forming characteristic mammospheres near the vessel; metastatic epithelial cells elongating and aligning with the vessel structures on the right side. Illustration corresponds with fluorescent light microscopy (**B**–**D**) and SEM images beneath (**E**–**F**). (**B**) Normal, healthy breast cells (HMEC) maintain their characteristic round shape in the presence of the endothelium, whereas the (**D**,**F**) metastatic MDA-MB-231 cells deform, elongate, and tightly adhere to the vessel structure. (**B**) White arrows indicate major axis length of epithelial cells. Healthy breast cells do not have a high affinity for directly contacting vessels, nor do they aggregate as much as (**C**,**E**) tumorigenic cell lines or elongate like (**D**,**F**) metastatic cell lines. Representative scanning electron microscopy images of epithelial (EPI) -endothelial (ENDO) co-cultures show EEC interaction phenotypes in HUVEC endothelial co-cultures with (**E**) tumorigenic non-metastatic, and (**F**) metastatic cells lines. Endothelial tubes are outlined with dotted lines.

Primary, tumorigenic non-metastatic, and metastatic breast epithelial cells were added to human endothelial tubes formed within a Matrigel® matrix. Observation of epithelial-endothelial cell (EEC) interaction phenotypes revealed stark differences (Fig. 1). Primary epithelial cell lines (HMEC, MCF-10A) preferentially interact with other epithelial cells, exhibiting minimal interactions with the endothelium. Tumorigenic cell lines (SkBr3, MCF-7) form large tumor spheroids with limited interaction with the endothelium. Metastatic cell lines (MDA-MB-468, MDA-MB-231) tend to align along the vessel, elongate, and potentially invade the endothelium. See Table 1 for further descriptions of the cell lines used.

**Table 1:** Description of cell lines used.

Cell line descriptions	
Cell Line	Description
HUVEC	Human primary umbilical vein endothelial cells
HMEC	Human breast epithelial cell line, isolated from a healthy patient
MCF-10A	Human breast epithelial cell line, isolated from a patient with fibrocystic disease
SkBr3	Human breast adenocarcinoma cancer cell line
MCF-7	Human breast epithelial, tumorigenic, non-metastatic, cancer cell line
MDA-MB-468	Human breast epithelial, metastatic, cancer cell line
MDA-MB-231	Human breast adenocarcinoma cancer cell line, derived from metastatic site via pleural effusion

Metastatic cells (Fig. 1A,D,F) display a higher degree of interaction with the endothelium, aligning and elongating along the vessels as compared to control (Fig. 1A,B). Figure 1B–D show representative examples of immunofluorescence images that highlight the dramatic change in shape that the metastatic cells undergo when in contact with the endothelial tubes which is captured in further detail by SEM images of non-metastatic (Fig. 1E) vs metastatic (Fig. 1F) co-culture interactions.

**Figure 2:**
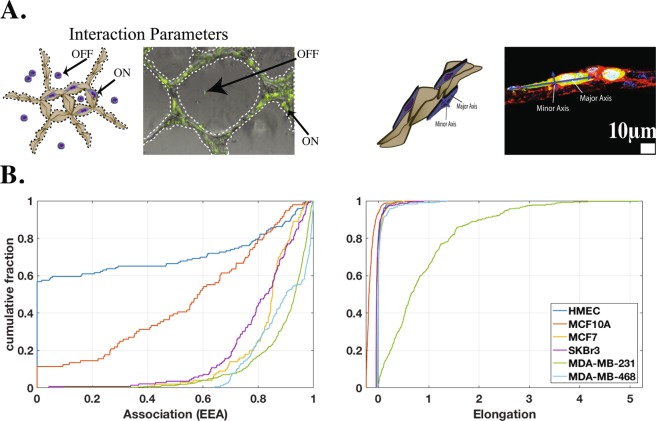
(**A**) Representative composite bright field and fluorescence microscopy images of co-cultures. Epithelial cell lines were labeled with CFSE (green). (**B**) Cumulative distribution of elongation and association data. Metastatic cells exhibited increased interaction with and elongation along the endothelial tubes compared to other cell types, in particular MDA-MB-231 cells.

To better understand these interactions, we developed two quantifiable interaction parameters: (1) EEA (association) and (2) elongation. EEA indicates the degree of interaction between the cell types by quantifying the relative fraction of cells that are physically interacting with the endothelial tubes (Fig. 2A). Elongation is used to quantify the cellular deformation that occurs when the epithelial cells come in contact with the endothelium. Figure 2B summarizes quantification results for the association and elongation. Calculations used for quantification analyses can be found in the Quantification of interaction parameters section.

### Association, elongation, and 2-Parameter Index

Figure 2A illustrates the quantification criteria for the two interaction parameters. The EEA metric provides a measure of the relative quantity of epithelial cells interacting with the endothelium. For each individual cell, association is a binary state (i.e. “on” or “off”). An epithelial cell’s interaction with the endothelium is determined to be “on” or “associated” with the endothelium if it is physically in contact with any part of the endothelial tubes in the co-culture. In microscopic images, epithelial cell boundaries can be contiguous and indistinguishable. Due to difficulty in determining cell boundaries, and also to simplify quantification, epithelial cells were labeled with CFSE before being introduced in the co-culture; fluorescence was then used to determine the ratio of epithelial cells “on” the endothelial tubes to the total number of epithelial cells. By using several images from varying regions across several co-cultures, we are able to obtain a large enough sample such that this ratio is representative. See the Quantification of interaction parameters section for further details.

Cells with a low EEA have little interaction (i.e. primary epithelial cells – HMEC data in Fig. 2B), while cells with a high EEA exhibit a high degree of interaction with the endothelium (i.e. metastatic cells – MDA-MB data in Fig. 2B). As discussed previously, primary cells show minimal interaction with the endothelium. The highly metastatic MDA-MB-231 cells show the highest degree of interaction with the endothelium, which was statistically significant compared to both primary (p < 0.0001) and tumorigenic (p < 0.0001) cell lines. The moderately metastatic cell line, MDA-MB-468, showed slightly less interaction with the endothelium compared to the highly metastatic MDA-MB-231 cells, suggesting that increased interaction with the endothelium may correlate with increased invasive capacity. These results support a trend indicating that metastatic cells lines greatly interact with the endothelium. Furthermore, the EEA metric easily distinguishes the tumorigenic and metastatic cells from the normal cell populations. Measurement of EEA could shed light onto the invasive potential of a population of cells.

Unlike association, elongation is not a binary “on/off” measurement for each cell. The elongation metric quantifies the change in shape of epithelial cells while interacting with the endothelium. This is done by normalizing the inverse circularity value of each cell “on” by the average value of those “off” for each cell type. This normalization ensures that any elongation behavior we observe is purely due to the presence of the endothelium and not an intrinsic property of any particular cell line. When “off” the endothelium, the breast epithelial cells appear rounded with no distinct cellular polarity, having an inverse circularity near 1.0 for all cell lines, independent of metastatic potential. Normal and tumorigenic cells have little to no change in morphology when in contact with the endothelium, maintaining this morphology or becoming more rounded, indicated by an elongation distribution very close to zero, with some slightly negative values (Fig. 1B). A negative elongation characterizes cells that are more rounded when interacting with the endothelium. Unlike the normal (p < 0.001) and tumorigenic (p < 0.001) cell types, metastatic cells undergo a significant deformation from rounded to spindle shaped when in contact with the endothelium. This is observed in the cumulative distribution function of elongation values greater than zero in Fig. 2B. The highly metastatic MDA-MB-231 cells have a much wider distribution of elongations, whereas the other cell types are more concentrated near zero (spherical). This cellular deformation may be required for successful intravasation and may be a unique property of metastatic cells.

**Figure 3:**
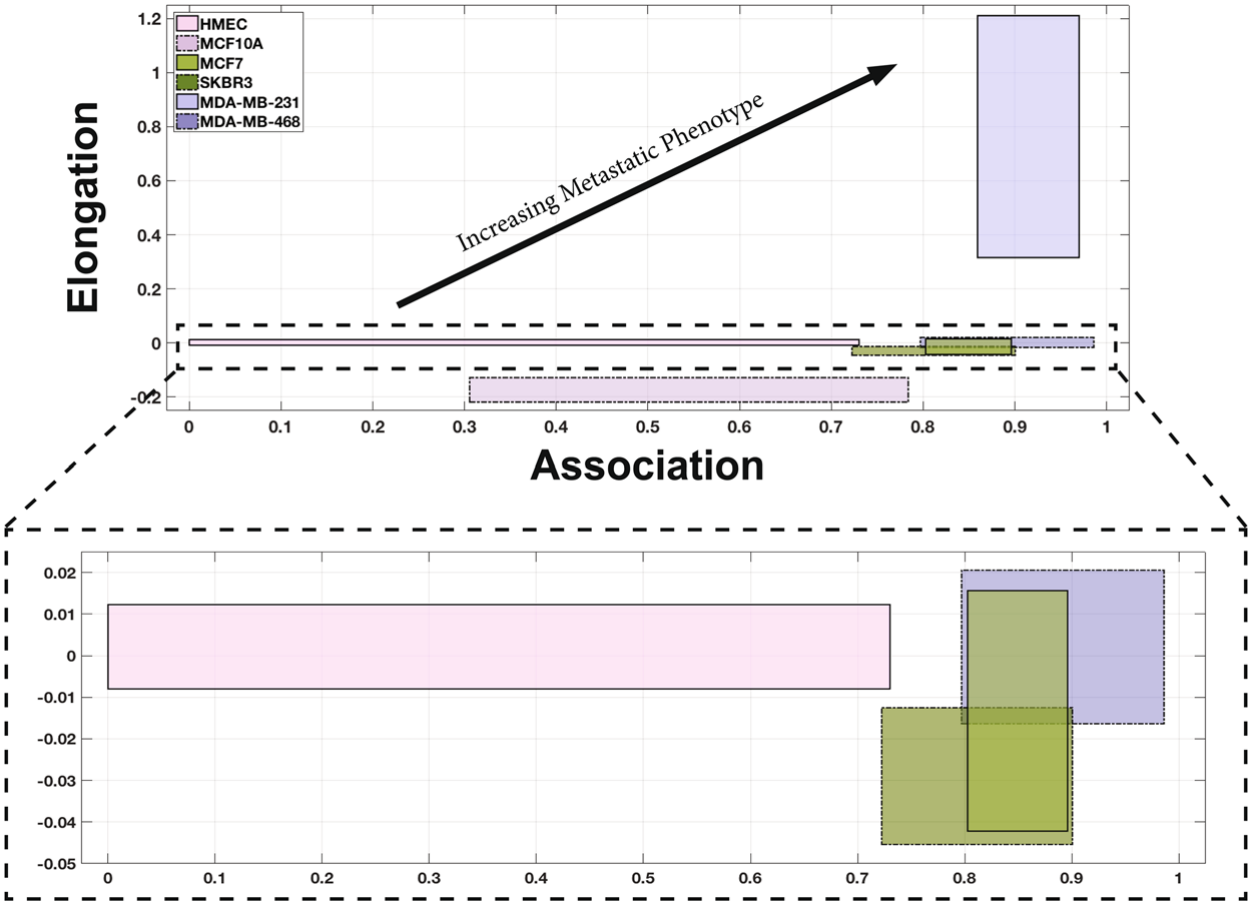
The association and elongation interaction parameters can be used together (2-parameter index) as a predictive model for identifying metastatic capacity based on the behaviors of cells in co-culture. The graph shows how the measurement of EPI-ENDO association and elongation parameters can significantly distinguish the metastatic cells (blue boxes) from the tumorigenic (green boxes) and primary (pink boxes) epithelial cells. The boxes bound the 1st to 3rd quartiles of the association (horizontal axis) and the elongation (vertical axis). The region near the horizontal axis has been expanded to identify the data more clearly.

Using the results from the interaction parameters summarized in Fig. 2B, we created a 2-parameter metastatic index. The graph in Fig. 3 illustrates the relationship between association and elongation parameters for the cell lines. In this figure, each cell line is characterized by a region bound by the 1st and 3rd quartiles of the two parameters. The association parameter is plotted on the horizontal axis, and elongation on the vertical axis. From this graphical representation, which we refer to as the 2-parameter index, emerge regions that can differentiate cell populations by invasive potential. As indicated by the arrow in Fig. 3, highly invasive cell types can be found closer to the upper right region of the graph, while cell types that are less likely to display invasive characteristics can be found closer to the lower left region.

### Invasive potential energy analysis

Metastatic cells have the ability to undergo large deformations when in contact with the endothelium, resulting in dramatic changes in morphology from rounded structures to flat fibroblast-like structures. This change in cellular morphology may provide an advantage for metastatic invasion. Indeed, studies have shown that the adoption of a flat, spindle-like fibroblast architecture aids cells during metastatic invasion^11^.

**Figure 4:**
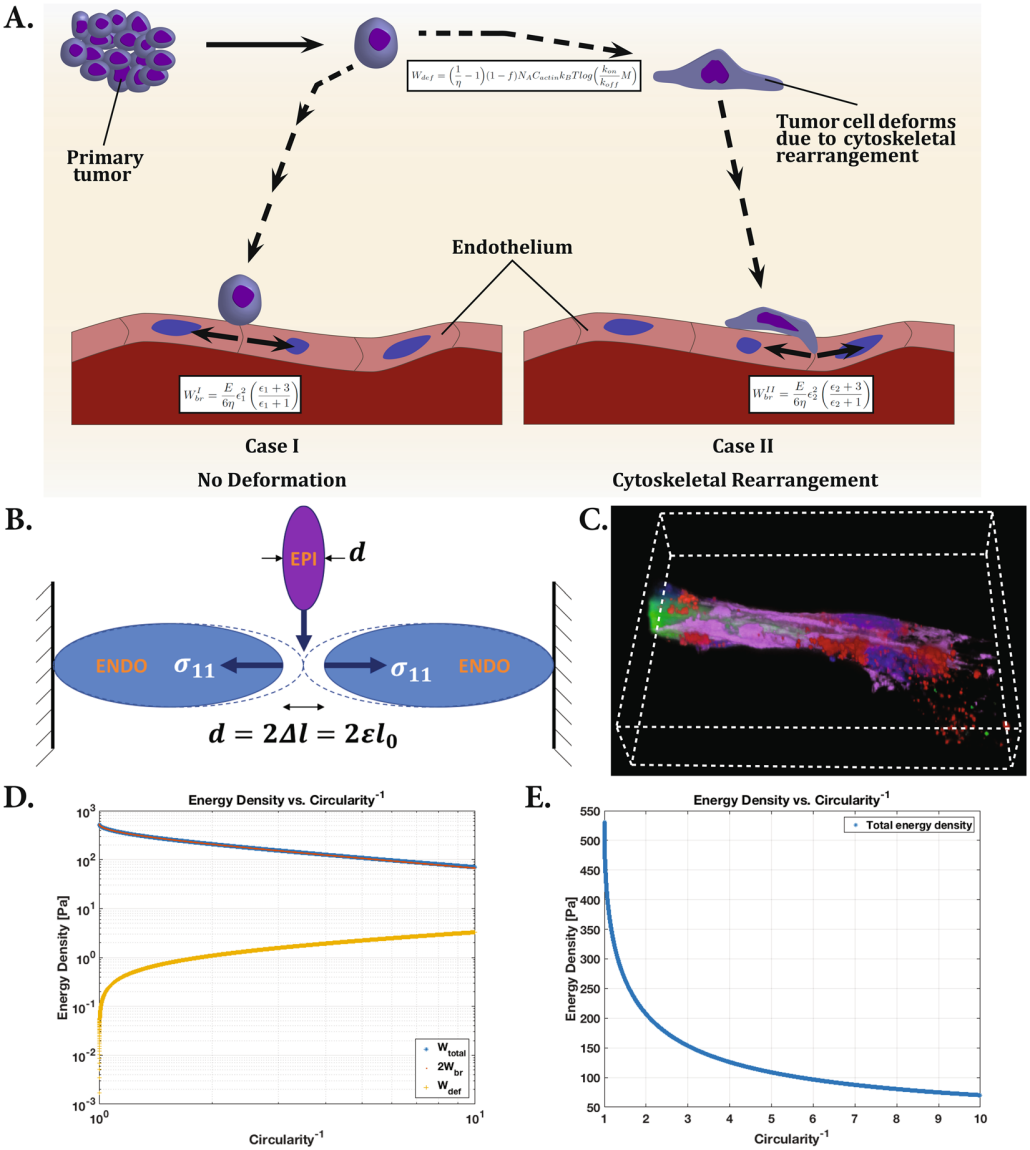
(**A**) Tumor cell invasion of the endothelium, beginning with separation from primary tumor, migration to endothelium, and intravasation. Case I illustrates invasion without deformation of the tumor cell, while case II illustrates tumor cell invasion after cytoskeletal rearrangement and deformation of the cell. The equations describe energy expenditure by the tumor cell in undergoing deformation and endothelial barrier breech. (**B**) Schematic of model for endothelial deformation. (**C**) Fluorescence microscopy image of the co-culture model, capturing a tumor cell contained within an endothelial tube. (**D**) Log-log plot of the epithelial cell energy density components as a function of inverse circularity (measure of deformation). The deformation energy is orders of magnitude smaller than the energy required to breech the endothelium. The relatively minuscule energy investment prior to breeching the endothelium can save a significant amount of energy overall. (**E**) Linear plot of total energy density versus inverse circularity. As the cell elongates, the energy expenditure of intravasation decreases rapidly.

The interaction parameters give us insight into the likelihood of a specific cell type having metastatic characteristics. This likelihood is derived from the cells’ propensity to lengthen and align with the endothelial tubes. We hypothesize that the lengthening and alignment of the tumor epithelial cells along the endothelial tubes allows the tumor cells to penetrate the tubes and traverse the vascular network. This has been demonstrated in our co-culture model. Figure 4C shows a fluorescence microscopy image from our co-culture model, in which a tumor cell has altered its morphology and successfully invaded the endothelial network. This method of intravasation provides a more mechanically efficient means for the tumor cells to metastasize through either the blood or lymphatic vasculature.

Figure 1B,D shows confocal fluorescence microscopy images of behavior of HMEC and MDA-MB-231 co-cultures. The HMEC maintains its characteristic round shape in co-culture, while the MDA-MB-231 cells undergo cellular deformation in the co-culture. There is some energy cost for the cell to rearrange its cytoskeletal composition in order to deform. This cost is directly related to how much the cell elongates, or deforms.

Consider a tumor cell leveraging its internal energy to breach the vascular wall. Figure 4A is a cartoon illustration of metastatic progression, beginning with separation from the primary tumor, migrating and ultimately entering the endothelium. We assume that the epithelial cell traverses the vessel wall at a capillary junction between two adjoining cells because this is more energetically efficient than burrowing through an endothelial cell. Metastatic cells breeching the endothelium have been shown to force their way between junctions, similar to diapedesis, but penetrating the vessel wall via mechanical leverage^33^. Figure 4A illustrates this scenario in two possible cases. In case I, a spherical tumor cell is modeled crossing a narrow junction between two endothelial cells. In case II, the tumor cell deforms itself by rearranging its cytoskeletal structure prior to crossing the endothelial cell-cell junction, as we have observed experimentally from the highly metastatic MDA-MB-231 breast epithelial cells co-cultured with HUVEC cells. Figure 4B is a geometrically accurate representation of our model. Both epithelial and endothelial cells are modeled as prolate spheroids (ellipsoid symmetric about the major axis). Deformation of the epithelial cell will alter *d*, the minor axis length, equal to the twice the deformation of each of the endothelial cells. For case II, we consider a range of deformation, from zero deformation (equivalent to case I), to an inverse circularity value of 10 – beyond the largest deformation observed experimentally (6.31). The cytoskeletal rearrangement of the tumor cells is modeled based on cytoskeletal rearrangement mechanics observed in neutrophils by Yap *et al*.^34^, which have been analyzed more extensively due to their ability and need to quickly rearrange their cytoskeleton to fit into tight junctions. We define case I, ${W}^{I}={W}_{{C}^{-1}=1}$, and case II, ${W}^{II}={W}_{{C}^{-1} > 1}$, representing a range of energy density.

As one might expect, the energetic analysis indicates that the energy density requirement for vessel breech in case I (${W}_{br}^{I}$) is larger than that of case II (${W}_{br}^{II}$). In both cases, the epithelial cell must expend energy to breach the endothelial vessel wall by compressing the two adjoining endothelial cells during intravasation and again during extravasation, prior to finding a secondary tumor site (2*W*_*br*_). Using a simple model, we assume only the two adjoining endothelial cells forming the junction are compressed. Both epithelial and endothelial cells are modeled as spheroids with geometric dimensions taken from our experimental measurements. The analysis shows that energy cost to the epithelial cell in the deformation/reformation process (*W*_*def*_) is minuscule in comparison (less than 3% of the energetic cost of breaching the endothelium).

$${W}^{I} > {W}^{II}$$

$$2{W}_{br}^{I} > 2{W}_{br}^{II}+{W}_{def}$$

For the purposes of this energetic analysis, we observe the following assumptions:

1. Epithelial cell traverses vessel wall at a capillary junction between two adjoining endothelial cells
2. Only the two adjoining endothelial cells forming the junction undergo uniaxial compression along their major axis
3. All cells are modeled as incompressible neo-Hookean (hyperelastic) solids; (*ν* = 0.5 — Poisson’s ratio)
4. Endothelial cell axial stresses are much larger than shear stresses
5. Actin filament concentration and cross-links in epithelial cell cytoskeleton are similar to that of neutrophils
6. Circularity and relative f-actin concentration relation in cells is similar to that of neutrophils
7. Relative f-actin content in an epithelial cell is proportional to its cross-sectional area
8. Epithelial cell volume remains constant throughout cellular deformation and vessel wall breech

In reality, deformation of endothelial cells is transmitted across multiple cells, both axially and also tangentially in the vessel. Furthermore, tumor cells have been observed breeching vessels at junctions involving three or four epithelial cells and also using nanoscale tethers that may aide in invasion^28^. For the purpose of this simplified model, we assume the endothelial cell deformation is confined to the two endothelial cells forming a junction, and the strain is distributed along the axis of the vessel. Given these assumptions, we can derive a strain energy density function (*W*_*br*_), related to the deformation of the endothelial cells.

1 $${\eta }_{br}{W}_{br}=\frac{G}{2}({\lambda }_{1}^{2}+{\lambda }_{2}^{2}+{\lambda }_{3}^{2}-3)$$

*λ* represents the principal stretches, with 1 being in the axial direction, and *G* is the shear modulus. In an ideal case, the energy exerted by the epithelial cell to deform the endothelial cells would equal the deformation energy of the endothelial cell, however due to mechanical/thermodynamic losses, we introduce an efficiency term, *η*_*br*_. By implementing the incompressible and uniaxial stretch assumptions, we reduce the strain energy density to the following:

$${\lambda }_{1}^{2}{\lambda }_{2}^{2}{\lambda }_{3}^{2}=1\,({\rm{Incompressible}}\,{\rm{solid}})$$

$${\lambda }_{1}=\lambda ;\,{\lambda }_{2}={\lambda }_{3}={\lambda }^{-\frac{1}{2}}({\rm{uniaxial}}\,{\rm{compression}})$$

2 $${W}_{br}=\frac{G}{2{\eta }_{br}}({\lambda }^{2}+\frac{2}{\lambda }-3)=\frac{G}{2\lambda }{(\lambda -1)}^{2}(\lambda +2)$$

Rewriting in terms of the principal strain (*ε*^11^ = *ε* = *λ* − 1) instead of the principal stretch, we obtain the following general expression for both cases:

3 $${W}_{br}=\frac{G}{2{\eta }_{br}}{\varepsilon }^{2}(\frac{\varepsilon +3}{\varepsilon +1})$$

For an isotropic, linear, elastic material, the shear modulus (*G*) obeys the following relation:

4 $$G=\frac{E}{2(1+\nu )}$$

where E is the elastic modulus. For an incompressible material, *ν* = 0.5, and this equation reduces to

5 $$G=\frac{E}{3}$$

We use the HUVEC elastic modulus measured (using atomic force microscopy) by Sato *et al*.^35^, giving us our final energy density expression for breaching the endothelial vessel.

6 $${W}_{br}=\frac{E}{6{\eta }_{br}}{\varepsilon }^{2}(\frac{\varepsilon +3}{\varepsilon +1})$$

To find the strain *ε*, we must relate the geometric dimensions of the epithelial cells for the spherical and deformed cells. We obtain a relationship between the sphere radius (*r*) and the spheroid semi-major/minor axes (*a* and *b*) by equating the volume for a sphere and a prolate spheroid, given that we assume the cell volume does not change during cytoskeletal rearrangement. The dimensions used for epithelial and endothelial cells are median values captured in the co-culture model.

7 $${V}_{cell}=\frac{4}{3}\pi {r}^{3}=\frac{4}{3}\pi a{b}^{2}\to constant$$

Equation 7 gives us the relationship $a=\frac{{r}^{3}}{{b}^{2}}$, allowing us to express the projected area in terms of *r* and *b*

8 $$A=\pi {r}^{2}(\frac{r}{b})$$

Also, based on our assumption that the cell deformation is similar to that of neutrophils, we can derive a second expression for circularity (*C*) of the ellipse, which is calculated from epithelial projected area (*A*) and perimeter (*P*) measurements:

9 $$C=\frac{4\pi A}{{P}^{2}}=\frac{4{\pi }^{2}ab}{{(\pi (3(a+b)-\sqrt{(3a+b)(a+3b)}))}^{2}}$$

Substituting for *a*, we obtain a expression relating *C* to $(\frac{r}{b})$.

10 $$C=\frac{4{(\frac{r}{b})}^{3}}{{[3({(\frac{r}{b})}^{3}+1)-\sqrt{(3{(\frac{r}{b})}^{3}+1)({(\frac{r}{b})}^{3}+3)}]}^{2}}$$

From Fig. 4B, note that the endothelial cell is compressed by a length equal to the semi-minor axis of the epithelial cell. From our experimental measurements in the co-culture, we found the endothelial HUVEC cell length to be, *l* = 30.97 ± 13.34 *μm*, while the healthy epithelial HMEC cells have an average diameter of *d*_*I*_ = 2*r* = 10.62 ± 2.37 *μm*.

11 $${\varepsilon }_{I}=\frac{{d}_{I}/2}{l}=\frac{5.31\,\mu m}{30.97\,\mu m}=0.1715,$$

giving us the strain for case I (no deformation of epithelial cell). Similarly for case II,

12 $${\varepsilon }_{II}=\frac{{d}_{II}/2}{l}={(\frac{r}{b})}^{-1}{\varepsilon }_{I}$$

Solving equations 10 and 12 numerically, we substitute into equation 6 to obtain *W*_*br*_ as a function of *C*, for $C,\frac{r}{b}\in (0,1]$.

Next, we calculate the energy density expended rearranging the cytoskeletal structure of the cell. Yap *et al*. measured both actin filament concentration and circularity of the cell membrane, allowing us to deduce a relationship between these two properties. A relative f-actin content of 80% corresponding with a circularity of 0.7 (relative diameter of 60%) is used as a benchmark, based on experimental data from Yap^34^. By assuming that relative f-actin content is directly proportional to the cross-sectional area of the epithelial cell, and using the aforementioned relative f-actin content to circularity benchmark, we can use equations 8 and 10 to numerically calculate the f-actin concentration as a function of the cell’s circularity (or inverse circularity), *f*_*act*_ = *f*(*C*^−1^).

Cytoskeletal rearrangement involves breaking of actin-filament bonds, which release chemical energy. After passing through the endothelial vessel junction, the cancer cell will use that energy to reform the actin-bonds and return to its original cytoskeletal structure. Due to thermodynamic inefficiency, the system must expend more energy than it released from its bonds. The g-actin monomer binding free energy can be expressed as^16^

13 $${\rm{\Delta }}{G}_{B}={k}_{B}Tlog(\frac{{k}_{on}}{{k}_{off}}M)$$

Multiplying this monomer binding free energy by the actin concentration of the cell (*C*_*act*_), the Avogadro constant (*N*_*a*_), and the fraction of bonds actually broken (1 − *f*_*act*_, where *f* is the relative f-actin content described in assumptions above), we obtain the energy density of the bonds in the entire cell (*W*_*def*_).

14 $${W}_{act}=(1-{f}_{act}){N}_{A}{C}_{act}{k}_{B}Tlog(\frac{{k}_{on}}{{k}_{off}}M)$$

Without considering for losses, the cell would gain and lose *W*_*act*_ from breaking and reforming the actin bonds, with a net *W*_*def*_ = 0. We consider these losses by introducing the efficiency (*η*_*act*_) of the actin polymerization. The difference in energy density due to deformation is

15 $${W}_{def}=\frac{{W}_{act}}{{\eta }_{act}}-{W}_{act}=(\frac{1}{{\eta }_{act}}-1){W}_{act}$$

This gives us a final deformation energy density expression:

16 $${W}_{def}=(\frac{1}{{\eta }_{act}}-1)(1-{f}_{act}){N}_{A}{C}_{act}{k}_{B}Tlog(\frac{{k}_{on}}{{k}_{off}}M)$$

With expressions for *W*_*def*_ and *W*_*br*_, we can apply the appropriate values from Table 2 to find the energy density for the entire range of deformation. We find that ${W}^{I}=2{W}_{br}^{I}=530\,Pa$ (*C*^−1^ = 1, no deformation).

**Table 2:** Parameters used in energy analysis calculations.

Energy analysis table of parameters		
Parameter	Value	
*C*_*act*_	50 *μM*	f-actin concentration
*M*	10 *μM*	g-actin monomer concentration
*N*_*A*_	6.022 × 10^23^ *mol*^−1^	Avogadro’s constant
*T*	310 *K*	temperature
*η*_*br*_	0.15	mechanical efficiency
*η*_*act*_	0.15	polymerization efficiency
${f}_{act}^{=0.7}$	0.8	relative f-actin content
*k*_*B*_	1.381 × 10^−23^ *JK*^−1^	Boltzmann constant
*k*_*on*_	11.6 *μM*^−1^*s*^−1^	polymerization rate constant
*k*_*off*_	1 *s*^−1^	depolymerization rate constant
*E*	10 *kPa*	elastic modulus
*ε*_*I*_	0.1715	case I principal strain

The energy density required to deform the epithelial cells is orders of magnitude smaller than the breeching energy (Fig. 4D). Therefore, the total energy density required of case I is much larger than that of case II for any appreciable amount of deformation. Taking the median observed inverse circularity value for the highly metastatic MDA-MB-231 cell line, ${C}_{50{\rm{ \% }}}^{-1}=1.68$, corresponds to *W*^*II*^ = 239 *Pa*, or 55% less energy expended than case I. If we expand the inverse circularity range of this cell line to the 1st and 3rd quartiles, we have a range of energy density between 187 *Pa* and 296 *Pa*.

An epithelial cell that is able to deform prior to traversing the barrier expends about 55% less energy, for *C*^−1^ = 1.68). The required energy density is shown in Fig. 4D,E as a function of the full range of inverse circularity of the epithelial cell shape. Increasing inverse circularity correlates to the deformation/elongation of the cell. As the cell elongates from its initial round shape, there is a rapid drop in the energy density requirement. As the cell continues to elongate, the energy density requirement is further reduced, but at a slower rate.

### Effect of cytoskeletal disruptor

Altering the cell’s morphology through cytoskeletal rearrangement appears to be a critical step leading to invasive potential. To further support our hypothesis of the role of cytoskeletal rearrangement, we tested the most invasive cell line, MDA-MB-231, in the co-culture system, along with docetaxel, a commonly used chemotherapeutic and cytoskeletal disruptor^36^. Specifically, a docetaxel nanoparticle was used to treat the cancer cells. The docetaxel nanoparticle is synthesized as reported by Kulkarni *et al*.^37^.

**Figure 5:**
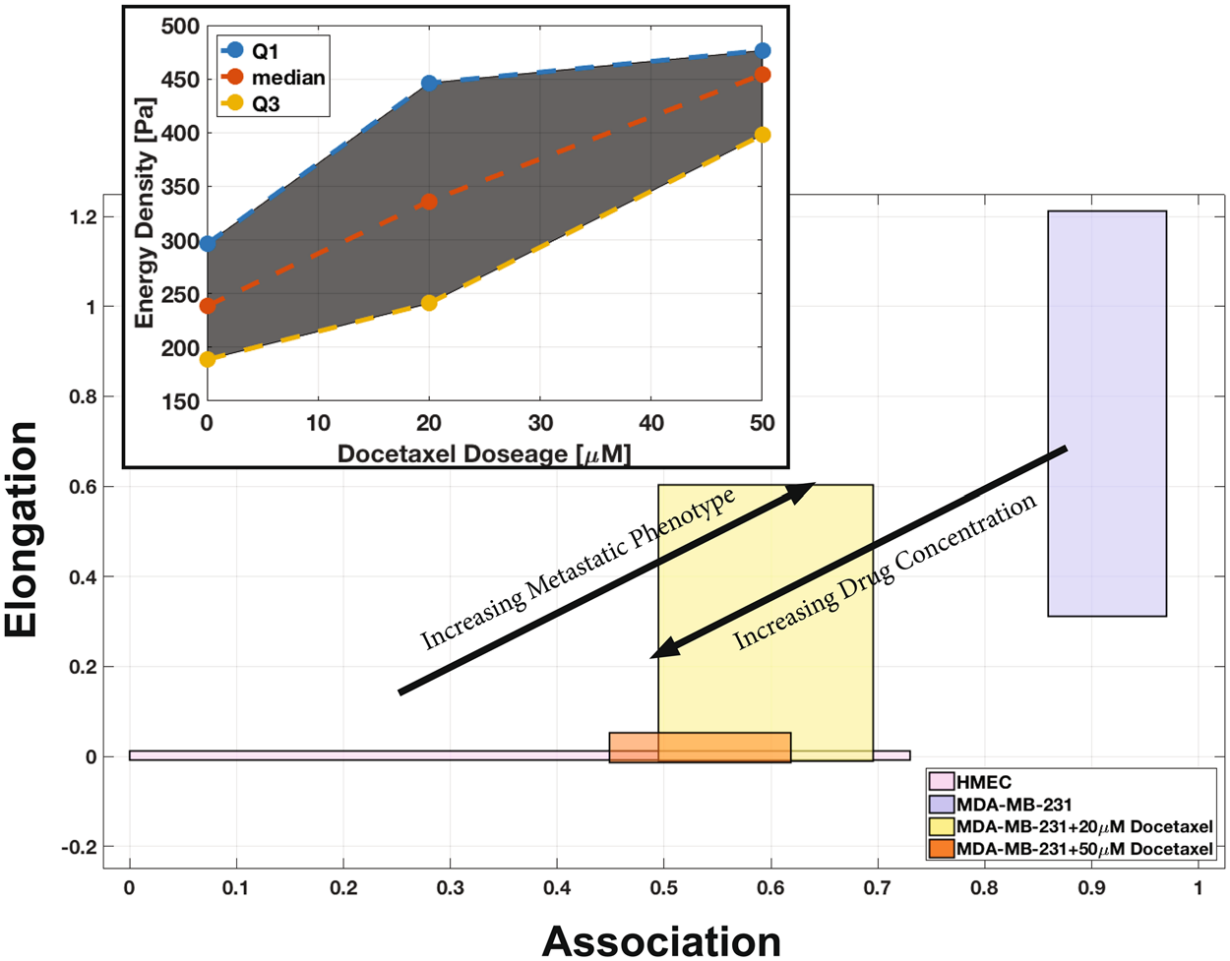
The 2-parameter index is again used to identify degree of metastatic phenotype based on the behaviors of cells in co-culture as illustrated in Fig. 3. However, this diagram illustrates the effect of cytoskeletal disruptor docetaxel on a known metastatic cell line, MDA-MB-231 in the co-culture. After treatment with 20 *μM* docetaxel (yellow box), the 2-parameter index of the MDA-MB-231 cells shift down and left, presenting with a less metastatic behavior (i.e. less association with the endothelium and less elongation). A higher concentration of 50 *μM* docetaxel treatment (orange box) produces a more dramatic shift, overlapping the 2-parameter index of the treated cells with that of healthy epithelial cells (pink box). The untreated MDA-MD-231 cell data is represented by the blue box. The inset panel shows the relationship between the energy expenditure required for the MDA-MB-231 cells to metastasize after docetaxel treatment. The shaded region bounds the 1st and 3rd quartiles.

Figure 5 shows the 2-parameter index comparison of the drug-treated metastatic cell lines with the untreated metastatic cell line and the normal primary cells. Interestingly, increasing the concentration of the drug shifts the 2-parameter index of the cell line from the invasive, upper right hand region of the graph where the control data lies, to the lower-left hand region of the graph where the primary cell line data lies. Furthermore, the shift in the 2-parameter index indicates dose-dependency of the cytoskeletal disruptor on the behavior of the epithelial cells. With increasing dosage of docetaxel, we observe an increasing reversal of the metastatic behavior in MDA-MB-231 cells. The untreated metastatic cells exhibit median association and elongation values of 0.93 and 0.66, respectively. When treated with 20 *μM* docetaxel, both median association and elongation drop to 0.58 and 0.16, respectively. When treated with 50 *μM* docetaxel, both median association and elongation further drop to 0.54 and −0.0018. This drop, particularly in the elongation with drug treatment, is much more drastic, approaching median elongation of HMEC (primary) cells (−0.0026). This suggests that inhibiting the cell’s ability to rearrange its cytoskeleton in order to alter its morphology, may prevent the cell from traversing the endothelial tube network.

From the observed elongation values of drug-treated metastatic cells, the inverse circularity is calculated, which also can be mapped to the strain energy density function, *W*, calculated previously (Fig. 4D,E). The metastatic cells treated with 20 *μM* docetaxel expend 336 *Pa*, 40% more energy than the non-treated cells. Treatment with 50 *μM* docetaxel lead to energy density expense of 454 *Pa*, 90% more energy than non-treated metastatic cells, but only 14% less than the normal HMEC cells. The inset plot in Fig. 5 shows the relationship between the drug dosage and energy requirement for the cells to metastasize. The drug-treated endothelial-epithelial interaction behavior is quantitatively more similar to the normal cell line, than to the untreated metastatic cell behavior.

## Discussion

The endothelial-epithelial co-culture model allows for observation of physical interactions between two cell types, providing insight into the dynamics between tumor cells and their environment. These interactions help highlight the key factors leading to cancer metastasis. In conjunction with mathematical models, we can better understand the physical, biochemical, and physiological interplay between the components. We hypothesize that cancer cell types that are prone to display metastatic behavior interact differently with the endothelium than cells that are not; in particular, these metastatic cells are attracted to vessels and alter their morphology to provide a more mechanically efficient means of migration in their quest to invade the endothelium.

The epithelial cell’s mechanical properties are important for understanding how it responds to external stimuli, interaction with the environment, and migration. It has been demonstrated that metastatic cells are generally more compliant than non-turmorogenic cells^38,39^. Evidence suggests that this may be due to changes in the structure of cytoskeletal proteins, primarily filamentous actin^33,38,40,41^. Additionally, there is evidence to suggest that cross-talk between cancer epithelial cells and the microenvironment will alter the mechanical properties of both the tumor cells, as well as the microenvironment, such that it is favorable for invasion^41–43^.

Evidence of epithelial cell changes in morphology was observed in our mathematical model, as well as through experimental measurements in the epithelial-endothelial co-culture model system. By examining the interactions between the endothelium and epithelial cells of known metastatic behavior in the co-culture model, we were able to observe phenotypic changes and associate the changes with cellular migration behavior. In particular, two phenotypic differences were observed to have a marked difference between metastatic and non-metastatic tumor cell lines.

Quantification of several EEC interaction phenotypes has led to identification of two parameters that characterize the interactions of tumor cells with the endothelium. The first is the degree of epithelial cell association with the endothelial tubes and the second is elongation of the epithelial cell body. These parameters together allow us to isolate and categorize tumor types based on their interaction phenotypes. Tumor cells with a higher degree of elongation and alignment along the endothelial vessels may have a greater likelihood of breaching the endothelium and entering the vessel structures. The energetic analysis indicates that such cytoskeletal rearrangement may play a role in the deformation of the tumor cells, presumably reducing the energy required to pry apart the endothelial cell-cell junctions.

It appears that association or alignment with the endothelium is a prerequisite for elongation. This is highlighted by the lack of an observable difference in the elongation of clustered or isolated epithelial cells that are not in physical contact with the endothelial tubes. This suggests that some mechanical stimuli must “prime” the tumor cells to elongate. Experimental observations show that cellular elongation can vary greatly, depending on the epithelial cell type. The quantified elongation measurements give us a range for calculating energy density expended in traversing the endothelium. For the metastatic MDA-MB-231 cell line, a median elongation value of 0.66 (*C*^−1^ = 1.68) corresponds to an energy (per unit volume) saving of 55%. This serves as a great advantage for tumor metastasis, providing a greater likelihood of intravasation. After these cells enter the circulation, they are able to travel to secondary sites to form new tumors.

Interestingly, drug studies using chemotherapeutic agent and cytoskeletal disruptor, docetaxel, show a reversal of the metastatic phenotypic changes. The elongation and association parameters normally observed in metastatic breast cell lines tend to revert to values similar to that of less metastatic or even normal breast epithelial cell lines. Based on this shift, we surmise that the cell’s inability to alter its morphology via cytoskeletal rearrangement will make it much more difficult to invade the endothelium and metastasize, due to the larger energy required to breech the endothelial tubes. Treatment with 50 *μM* docetaxel resulted in interaction parameters corresponding to a 90% increase in the energy required for intravasation. Similarly, Mierke *et al*. also reports decreased cell invasion with actin polymerizing disruption^11,42^.

Therefore, it is possible that interaction parameters, which measure the degree of tumor cell elongation and alignment along vessel structures, can be used together to assess metastatic potential of a given tumor. The use of 3D EEC co-culture model systems allow for efficient measurement and quantification of these parameters. By applying mathematical energetic analyses, we can assess invasive potential, and begin to classify tumor types. In the future, we aim to extend the co-culture model to incorporate higher order elements of the tumor microenvironment. This co-culture coupled with energetic analyses may provide a new platform for assessing and quantifying invasive capacity of various tumor types.

## Methods

### Establishing an epithelial-endothelial co-culture

Figure 1A is a schematic overview of the co-culture model system experimental design. The model system was established using primary human umbilical vein endothelium cells (HUVEC) obtained from ATCC. Endothelial cells were plated in a laminin-rich basement membrane matrix (1:1 PBS:Matrigel®) for 4–24 hours, wherein the cells spontaneously formed vessel-like structures. A high-magnification SEM micrograph illustrates the laminin-rich basement membrane matrix fibers in which the cells were embedded. The matrix provides the scaffold on which the cells can establish a three-dimensional conformation. Following completion of tubulogenesis, epithelial cells were added to the culture and the cells were incubated together for 24 hours unless otherwise stated. Epithelial cells were labeled with carboxyfluorescein succinimidyl ester (CFSE) cell-impermeable dye to distinguish the two cell populations. The organotypic model system combines endothelial cells with epithelial cells of varying grades of tumorigenicity, ranging from normal primary cells to highly metastatic cell lines. HUVEC endothelial cells were co-cultured with Human Mammary Epithelial Cells (HMEC), a primary breast epithelial cell line; MCF-10A, a non-tumorigenic, fibrocystic cell line; MCF-7 and SKBR3, tumorigenic/non-metastatic breast epithelial cell lines; MDA-MB-468, tumorigenic, low metastatic breast epithelial cell line; and MDA-MB-231, tumorigenic, highly metastatic breast epithelial cell line. Representative images of 3D co-cultures which can be seen in Fig. 1B–D. All cultures were incubated for 24 hours followed by immunostaining with rhodamine phalloidin (Red) and counterstaining with DAPI (Blue).

### Cell culture

HUVEC cells (ATCC) were cultured on 0.1% gelatin in EBM-2 (Lonza) supplemented with EGM-2 bullet kit (Lonza) and 0.1% antibiotic/antimycotic (A/A) (Life Technologies). Primary human dermal microvascular blood and lymph endothelial cells, collected from plasma, were cultured on collagen (1:60) in MCDB 131 supplemented with 5% MVGS (Life Technologies), 1% L-alanyl-L-glutamine (Life Technologies), and 1% A/A. MDA-MB-231 (ATCC), MDA-MB-435 (ATCC), B16 (ATCC), MCF-7 (ATCC), LLC (ATCC), 4306 (ATCC), and 4412 cells (ATCC) were cultured in DMEM supplemented with 10% FBS and 1% A/A. MDA-MB-231 GFP were cultured with 10% FBS and 1% Geneticin (Life Technologies). MDA-MB-468 cells were cultured in DMEM supplemented with 5% FBS and 1% A/A. SKBr3 (ATCC) were cultured in McCoy’s 5A (Life Technologies) supplemented with 15% FBS and 1% A/A. HMEC (Life Technologies) and MCF-10A (ATCC) cells were cultured in MEBM (Lonza) supplemented with MEGM bullet kit (Lonza) and 1% A/A.

### Co-culture

Endothelial cells were plated in their respective media on a laminin-rich basement membrane matrix (1:1 PBS:Matrigel®), and incubated for 4–12 hours to allow for vessel formation. Epithelial cells were loaded with CellTraceTM CFSE according to manufacturers’ specifications. The cells were then added to the preformed vessels in their respective media in a 1:1 epithelial cell:endothelial cell ratio.

### Immunocytochemistry (ICC)

Samples were fixed with 4% PFA at RT for 15 minutes and washed with sodium borohydride (dissolved in PBS). Cells were stained with the following: rhodamine phalloidin (Life Technologies) (1:100), p-Akt(S473), p-ERK(1/2), p-FAK(Y925), *β*1 Integrin. Nuclei were counterstained with DAPI (Life Technologies).

### Scanning electron microscopy

Samples were fixed with 0.1 M sodium cacodylate (Sigma), 2% gluteraldehyde (Electron Microscopy Sciences), 3% paraformaldehyde (Electron Microscopy Sciences), 5% sucrose buffer (Sigma) and 1% osmium tetroxide (pH 7.4) (Electron Microscopy Sciences). The samples were then dried in increasing concentrations of high-grade ethanol, followed by critical point drying using Autosamdri 815 critical point dryer and sputter coated using Cressington 208HR sputter coater with Au or Pt/Pd. Imaging was done on a Jeol 5600LV SEM, Zeiss EVO SEM, or Zeiss FESEM Ultra55 microscope.

### Fluorescence microscopy imaging

Fluorescence imaging was performed on a Nikon Eclipse Ti camera (Nikon Instruments) with NIS-Elements Microscope Imaging Software (3.10). Confocal fluorescence imaging was done on a PerkinElmer Ultraview Spinning Disk Confocal Microscope with Velocity acquisition software and Hammamatsu ORCA-ER CCD camera. Contrast and brightness parameter adjustments were applied across the whole image or equally across all the comparison groups when necessary using NIS-Elements Microscope Imaging Software (3.10). The length, width, and nodal area of the vessel structures was measured in more than 200 images, as well as the elongation of more than 300 epithelial cells aligned along the endothelial vessel structures, using NIS-Elements software. Z-stack images were processed using the NIS-Elements Advanced Research deconvolution module to generate 3D reconstruction images.

### Drug treatment

MDA-MB-231 cells were serum deprived for 6–18 hours. After serum deprivation, the cells were treated with the docetaxel nanoparticle in complete media for 24 hours. Once treated for 24 hours, the cells were then used in the co-culture system.

### Statistical analysis

All statistical analyses were performed using Prism 6 (GraphPad). A Student’s t-test, one-way analysis of variance followed by Bonferroni’s test to compare different groups, or two-way analysis of variance was used to calculate statistical significance, with P-values < 0.05 considered as significant. For each experimental group analyzed, the data was collected over a minimum of 3 experiments with a minimum of 3 replicates per experiment. Representative images were taken in order to capture an adequate sample to be quantified.

For interaction we assessed the following number of images (4x magnification):

- HMEC: 146 images
- MCF10A: 96 images
- SKBR3: 140 images
- MCF-7: 100 images
- MDA-MB-468: 154 images
- MDA-MB-231: 301 images
- Total: 937 images
  For elongation we assessed the following number of images (20x magnification):
- HMEC: 25 images with 271 cells ON/114 cells OFF
- MCF10A: 17 images with 320 cells ON/360 cells OFF
- SKBR3: 17 images with 312 cells ON/74 cells OFF
- MCF-7: 16 images with 360 cells ON/113 cells OFF
- MDA-MB-468: 21 images with 298 cells ON/138 cells OFF
- MDA-MB-231: 20 images with 305 cells ON/167 cells OFF
- Total: 116 images

Docetaxel Study:

For interaction we assessed the following number of images (4x magnification):

- Control: 110 images
- 20 nM: 107 images
- 50 nM: 115 images
- Total: 332 images

For elongation we assessed the following number of images (20x magnification):

- Control: 6 images with 49 cells ON/25 cells OFF
- 20 nM: 6 images with 70 cells ON/66 cells OFF
- 50 nM: 5 images with 90 cells ON/67 cells OFF

### Quantification of interaction parameters

Strict metrics were developed and applied to maintain consistency of the analysis over several cell lines and experiments. The rules applied in this analysis are summarized below.

Epithelial-endothelial association (EEA) is defined by the following expression:

$$EEA=\frac{F{l}_{on}}{F{l}_{total}},$$

where ***Fl***_***total***_ is a measure of the total fluorescence of all epithelial cells in a given image, and ***Fl***_***on***_ is a measure of the fluorescence of only those epithelial cells in contact with the vessel.

The following assumptions and rules were applied to quantify the epithelial-endothelial association:

1. An epithelial cell in physical contact with the endothelial tube is considered an interacting cell, or as being “on” the vessel, while an epithelial cell not in physical contact with the endothelium is considered “off” the vessel.
2. Fluorescence intensity was assumed to be proportional to the number of cells, such that a higher intensity indicates a greater number of cells.
3. The vessels are outlined as illustrated in **(**Fig. 2A**)**. The fluorescence intensity measured within the outlined region represents the fluorescence intensity on the vessel and is an indirect measure of the number of interacting cells.
4. Total fluorescence is an indirect measure of the total number of epithelial cells present in an image. Total fluorescence is equal to the fluorescence “on” plus the fluorescence “off” the vessel.

The elongation parameter is calculated for each cell “on” the endothelium by the following expression:

$$Elong=\frac{{C}_{On}^{-1}-\overline{{C}_{Off}^{-1}}}{\overline{{C}_{Off}^{-1}}},$$

where *C*^−1^ denotes the inverse of the circularity of the cell shape.

The following assumptions and rules were used in calculating the elongation:

1. An epithelial cell in physical contact with the endothelial tube is considered an interacting cell, or as being “on” the vessel, while an epithelial cell not in physical contact with the endothelium is considered “off” the vessel.
2. The major axis (length, *a*) and minor axis (width, *b*) of cells “on” and “off” the vessel were measured as depicted in Fig. 2A.
3. The inverse of circularity was used as a measure of the elongation of epithelial cells:
  $${C}^{-1}=\frac{{P}^{2}}{4\pi A}=\frac{{(\pi (3(a+b)-\sqrt{(3a+b)(a+3b)}))}^{2}}{4{\pi }^{2}ab}$$
  *C*^−1^ ranges from 1 (perfect circle) to infinity.
4. ${C}_{Off}^{-1}$ gives a measure of the normal, baseline cellular morphology of a cell that has not deformed due to interaction with the endothelium, and $\overline{{C}_{Off}^{-1}}$, is the average elongation value of all “off” epithelial cells.
